# Editorial: Immune response to respiratory viruses and respiratory viral infections in susceptible populations

**DOI:** 10.3389/fmed.2023.1330265

**Published:** 2023-11-17

**Authors:** Paraskevi C. Fragkou, Dimitra Dimopoulou, Giulia De Angelis, Giulia Menchinelli, Roy F. Chemaly, Chrysanthi Skevaki

**Affiliations:** ^1^1st Department of Critical Care Medicine and Pulmonary Services, Evaggelismos General Hospital, Athens, Greece; ^2^European Society of Clinical Microbiology and Infectious Diseases (ESCMID) Study Group for Respiratory Viruses (ESGREV), Basel, Switzerland; ^3^2nd University Department of Pediatrics, Panagiotis & Aglaia Kyriakou Children's Hospital, Athens, Greece; ^4^Dipartimento di Scienze Biotecnologiche di Base, Cliniche Intensivologiche e Perioperatorie, Università Cattolica del Sacro Cuore, Rome, Italy; ^5^Dipartimento di Scienze di Laboratorio e Infettivologiche, Fondazione Policlinico Universitario A. Gemelli IRCCS, Rome, Italy; ^6^University of Texas MD Anderson Cancer Center, Houston, TX, United States; ^7^Institute of Laboratory Medicine, Universities of Giessen and Marburg Lung Center, Marburg, Germany

**Keywords:** respiratory viral infections, immunology, critical illness, immunodeficiency, immunosuppression

## Introduction

Respiratory viruses are ubiquitous pathogens that cause infections of varying severity depending on attributes of the host and the virus itself (1). Data from the influenza pandemic in 2009, seasonal influenza epidemics and, more recently, the COVID-19 pandemic, underlie the importance of certain host risk factors that are associated with severe viral infections (1). Besides primary and iatrogenic/secondary immunosuppression that constitutes well known risk factors for basically any infection, other conditions such as pregnancy, obesity, diabetes mellitus, hypertension, cardiovascular disease, asthma, chronic obstructive pulmonary disease and extremes of age have been associated with high mortality and morbidity from respiratory viral infections (1).

The pathogenesis of immune dysregulation that is triggered by these host factors remains largely inexplicable. Revealing the differences and deciphering the commonalities among these conditions that render the host susceptible to severe viral infections will lead us a step closer to the development of more individualized therapeutic targets and preventative strategies. It is, therefore, vital to congregate the available evidence (including recent advances) and highlight the current research gaps in order to attest potential future therapeutic and preventative options.

The current Research Topic aims to highlight interdisciplinary research approaches that explore the role of host risk factors in the pathogenesis, progress and outcome of respiratory viral infections. Ultimately, the scope of this topic is to generate evidence on improved management and prevention of viral infections in susceptible populations, by assembling current knowledge and addressing potential gaps in research.

## Cell culture model and diagnosis of respiratory viral infections

Cell culture models are essential laboratory tools for studying *in vitro* models of host-pathogen interactions and the molecular mechanisms that are involved in the pathogenesis of viral infections. Fukuyama et al. presented their study on the establishment of a new porcine bronchial epithelial cell line from the respiratory tract of a neonatal pig. Through their experiments they concluded that porcine bronchial cells may represent a useful *in vitro* tool to investigate treatments that both potentiate antiviral immunity in the respiratory epithelium of the porcine host and can regulate Toll Like Receptor (TLR) 3- and TLR4-mediated inflammatory injury in the porcine airway which protects the host against harmful immune over responses.

In clinical context, early diagnosis and severity stratification of respiratory viral infections remains challenging in many cases as diagnostic molecular assays are not readily available in all clinical settings and routinely used biomarkers, such as C-reactive protein and white blood cell count, are not sensitive and specific for viral infections (2). Research is increasingly focusing on molecules that can be potentially used as biomarkers with high sensitivity and specificity for viral infections. Herzog et al. conducted a literature review on the surface adhesion molecule on human myeloid cells CD169 (also known as Siglec1 or Sialoadhesin), as a candidate screening biomarker for viral diseases. The authors concluded that even though CD169 shows a promising potential as a biomarker in acute viral diseases so far, universal laboratory standards and methodological groundwork are imperative to generate comparable and reliable results on its diagnostic performance in the context of viral infections.

## Severe acute respiratory syndrome coronavirus 2 infection

Severe Acute Respiratory Syndrome Coronavirus 2 (SARSCoV-2) cell entry is achieved through binding of the surface Spike (S) protein into its main host receptor angiotensin converting enzyme 2 (ACE2) (3). SARS-CoV-2 triggers inflammatory responses by affecting multiple cell types including type II alveolar epithelial cells and activating molecular pathways via its S protein, which have been shown to participate in the pathogenesis of COVID-19 (4–7). Al-Qahtani et al. showed that SARS-CoV-2 S protein suppressed inflammatory responses by decreasing the expression and secretion of interleukin (IL)-8, IL-6 and Tumor Necrosis Factor alpha (TNF-α) in alveolar type II cells during the early stages of infection, through activation of the PI3K/AKT pathway. The authors also suggested that at the early stages of the infection, S protein signals inhibit immune responses to the virus, which allows the propagation of the infection. Moreover, S protein signals in combination with TLR2 signals enhance Plasminogen Activator Inhibitor-1 (PAI-1) expression, which potentially affects the local coagulation cascade. The findings of this study propose the potential use of AKT/mTOR inhibitors for the regulation of inflammatory responses during SARS-CoV-2 infection.

SARS-CoV-2 infection has been considered as a trigger for autoimmune diseases through different mechanisms, including bystander activation, cross-reactivity, molecular mimicry, epitope spreading, and cryptic antigen unmasking (8). Tonutti et al. presented a case of anti- Melanoma differentiation antigen 5 (MDA5) syndrome with skin manifestations, constitutional symptoms, and cardiomyopathy following a confirmed SARS-CoV-2 infection in a 70-year-old Caucasian woman and then, they systematically searched for publications on inflammatory myositis associated with COVID-19, focusing on the anti-MDA5 syndrome. MDA5, a pattern recognition receptor, along with type I interferon (IFN) are important components of the immune response against viral infections. The activation of MDA5 induces the synthesis of type I IFN, which is inversely correlated with COVID-19 severity. A strong IFN signature has been associated with disease activity in various connective tissue diseases, including anti-MDA5 syndrome and might have protective effects against viral infections, including COVID-19. Finally, they concluded that SARS-CoV-2 may trigger the synthesis of autoantibodies and elicit an autoimmune response involved in inflammatory myositis pathogenesis, associated to the type I IFN rich molecular milieu promoted by the virus itself. In addition, Luo and Zhou identified common differentially expressed genes (DEGs) for COVID-19 and primary Sjogren's syndrome and performed enrichment and Protein-protein interaction network analysis. They found that COVID-19 and Sjogren's syndrome have common pathogenic mechanisms and pathways, that may be mediated by specific hub genes.

Diversity in response to SARS-CoV-2 exposure among elderly people may be related to differences in their innate immune responses (9). The gel-forming mucin 5B (MUC5B) is part of the mucus that covers the surface of the respiratory epithelium and plays a key role in the control of respiratory infections, the maintenance of immune homeostasis and the mucociliary clearance (10, 11). The decreased expression of MUC5B leads to declined mucociliary clearance, which has been correlated with aging (12). Moreover, constitutive expression of MUC5B levels is associated with the MUC5B promoter polymorphism rs35705950, while the high expressing T-allele is a risk factor for the non-infection-related aging lung disease, idiopathic pulmonary fibrosis (13). van Moorsel et al. investigated the association of MUC5B rs35705950 with severe COVID-19, in a retrospective candidate gene case-control study and the findings revealed that carriage of the T-allele of MUC5B rs35705950 may result in the protection from severe COVID-19, providing further evidence for the existence of trade-offs among optimal expression levels of MUC5B in the aging lung.

Alshammary et al. performed a systematic review and meta-analysis about the role of T-cell subsets and IL-10 levels among COVID-19 patients and their correlation with the disease severity and outcome. The results demonstrated that severe and non-survivor COVID-19 cases had lower counts of CD4/CD8 T-cells and higher levels of IL-10 compared to mild and survivor cases and the immunodepression following SARS-CoV-2 infection is possibly driven by IL-10. Also, they suggested that these clinical parameters may be reliable predictors of severity and mortality in COVID-19 patients.

Pinchera et al. evaluated the impact of mammalian Target of Rapamycin (mTOR) treatment on the evolution and outcome of SARS-CoV-2 infection in 371 kidney transplant recipients. No differences in the risk of acquiring SARS-CoV-2 infection were observed between the various immunosuppressive therapies. In contrast, patients who received mTOR inhibitors, as part of their immunosuppressive therapy, compared to other regimens had a lower chance of developing a moderate or severe disease. It is worth noting that multivariate analysis found that none of the variables considered showed a statistically significant impact regardless of the presence or absence of mTOR inhibitors. Therefore, mTOR inhibitors may be considered a possible treatment for COVID-19 in transplant and non-transplant patients, due to their potential antiviral or immunomodulatory properties.

In addition, it has been reported that vitamin D may be an important component in the prevention of respiratory tract infections, as it plays a signaling role in the modulation of the innate and adaptive immune response and immunoregulation (14). A systematic review and meta-analysis of randomized controlled trials was conducted by Kümmel et al. to assess the potential effects of vitamin D supplementation on the treatment and prevention of COVID-19 and its complications. The authors found that vitamin D supplementation is associated with a trend of decreased COVID-19-related mortality, shorter hospitalization, and less frequent admission to the Intensive Care Unit (ICU), especially in patients receiving repeated vitamin D doses, when vitamin D was given after the diagnosis of COVID-19.

Finally, acute respiratory distress syndrome (ARDS) in COVID-19 is triggered by hyperinflammation, indicating the need of immunosuppressive treatments. The Janus kinase (JAK) inhibitors, including Ruxolitinib, that block cytokine signaling pathways were found to improve outcome in hospitalized COVID-19 patients (15). Völkel et al. investigated the systemic effects of Ruxolitinib in critically ill COVID-19 patients by studying serum proteomes by mass spectrometry and cytokine array analyses at different time points after initiation of treatment. They demonstrated that the mechanism of action of Ruxolitinib in COVID-19 associated ARDS can be related to the SARS-CoV-2-infection and the effects of this drug as a modulator of T-cells.

## Other respiratory viruses

Cigarette smoking has been associated with an increased risk of contracting acute respiratory infections, as well as increased risk of developing severe infections and infection-related adverse outcomes (16, 17). Studies have demonstrated the complex interplay between host immune responses to influenza A and cigarette smoking, and how the latter may lead to worse infection outcomes (17). However, data on the effects of cigarette smoking on influenza B infection are limited. Chavez et al. developed an animal model to study how cigarette smoking can affect the course and severity of influenza B infection. The authors demonstrated that cigarette smoke extract reduced the influenza-B specific antibodies without compromising their neutralizing potency. Although they did not find any association between cigarette smoke extract exposure and viral replication, there was a dose-dependent effect of increasing cigarette smoke extract concentrations on mortality, insinuating a potential role of cigarette smoking in influenza B infection-related adverse outcomes in humans.

Besides influenza, Respiratory Syncytial Virus (RSV) represents a major contributor of infection-related hospitalizations with significant mortality and morbidity rates among adults and children, and especially in infants, elderly and immunocompromised patients (18–20). Besides supportive care, specific treatment options for RSV include ribavirin, palivizumab and RSV-immune globulin (only available in the United States), but are reserved for severe cases or at high risk for severe disease, including immunocompromized patients (21). Bindernagel et al. presented their experience of using a single dose of ASCENIV (an intravenous immunoglobulin that is manufactured from blending normal plasma with plasma from donors that possess high antibody titers against RSV and other respiratory pathogens) in three cases of critically ill children of < 5 years old, with some form of immune dysregulation. According to the authors, all three cases improved following administration of ASCENIV, concluding however, that well designed randomized controlled trials are needed to investigate whether ASCENIV is safe and effective in RSV infection.

Increasing evidence showed that inflammasomes activated by viral pathogens play a key role in viral clearance and tissue injury recovery (22). Li et al. investigated the role of non-canonical inflammasomes in the context of human adenovirus (HAdV) infection, another important respiratory pathogen that may lead to severe pneumonia, especially amongst susceptible hosts. The researchers found that HAdV infection induce macrophage pyroptosis by triggering non-canonical inflammasome activation via a NF-kB-dependent manner. They also noted that caspase-4 and caspase-5 may represent potential biomarkers associated with the severity HAdV-related pneumonia. These results reveal a new pathogenetic perspective on the pathogenesis of HAdV-related inflammatory tissue damage, that warrant further investigation.

## Author contributions

PF: Conceptualization, Data curation, Investigation, Methodology, Writing—original draft, Writing—review & editing. DD: Conceptualization, Data curation, Investigation, Methodology, Writing—original draft, Writing—review & editing. GD: Methodology, Project administration, Supervision, Writing—review & editing. GM: Methodology, Project administration, Supervision, Writing—review & editing. RC: Methodology, Project administration, Supervision, Writing—review & editing. CS: Methodology, Project administration, Supervision, Validation, Visualization, Writing—review & editing.
